# Fluid intelligence, social cognition, and perspective changing abilities as pointers of psychosocial adaptation

**DOI:** 10.3389/fnhum.2013.00287

**Published:** 2013-06-18

**Authors:** David Huepe, Natalia Salas

**Affiliations:** ^1^Laboratory of Cognitive and Social Neuroscience (LaNCyS), UDP-INECO Foundation Core on Neuroscience (UIFCoN), Universidad Diego PortalesSantiago de Chile; ^2^Faculty of Education, Cognitive Development Center, Universidad Diego PortalesSantiago de Chile

The prefrontal cortex in human brain is the main area that is related to the capacity for establishing relationships to others and with your environment. It is also the core for the development of superior mental functions such as plan and motor outcome, cognitive, affective, and social behavior across time (Kolb et al., 2012). Some cognitive functions related to this lobule include fluid intelligence (FI), social cognition (SC), and perspective changing abilities (PCA), which are necessary for adaptation to social contexts and solving problems in new situations (Barkley, 2001; Crisp and Meleady, 2012). These abilities, in turn, appear to be dependent on contextual keys, thus requiring flexibility, which is associated with frontal lobe functioning (Nestor et al., 2013; Pfeifer and Peake, 2012), particularly, in the case of PCA some areas are specifically related to the prefrontal cortex such as Brodmann area 10 (BA10) (Buckner and Carroll, 2007). In this opinion paper, we propose a model that integrates these components (FI, SC, and PCA) as indicators of psychosocial adaptation (PSA) in contexts of social vulnerability or diminished social/cultural conditions, in contrast to contributions in neurosciences made from evidence of patients with brain damage or psychiatric disorders.

PSA, defined as the quality of social life and subjective well-being of an individual in context (Bishop et al., 2008; Cox et al., 2010), is relevant for proper development. Research suggests that the prefrontal cortex plays a major role in adaptation, given its involvement in behavioral flexibility, executive functions, FI, and SC (Van Horn et al., 2012; Waters-Wood et al., 2012). Similarly, SC tasks, FI (Duncan et al., 1995; Roca et al., 2010) and cognitive flexibility have been associated with this area (Shamay-Tsoory et al., 2009; Larquet et al., 2010). Damage or alterations in the frontal lobe, have a direct impact on these functions, mainly resulting in maladaptive behaviors. Likewise, deficits in maturation or development of the cortex have been associated with social behavior disorders (Schore, 2000; Kolb et al., 2012).

FI has been defined as the ability to think logically and to solve problems in new situations, regardless of the acquisition of knowledge (Cattell, 1963). This reflects the ability to reason and to think abstractly in contrast to what is called crystallized intelligence (Cattell, 1967), which depends on cultural and academic learning. From a neuroanatomical viewpoint, FI has been associated with frontal lobe functions (Duncan et al., 1995). Injuries in this area affect the performance of these cognitive abilities (Roca et al., 2010; Woolgar et al., 2010). Besides, neuroimaging studies of FI have shown activation of frontal areas (Duncan et al., 2000; Bishop et al., 2008). There is consensus that the frontal lobe represents the neural basis of FI, but its association with cognitive flexibility and social behavior has not been studied until fairly recently. Support for the association between FI and cognitive flexibility and social behavior also comes from studies on frontal lobe lesions (Hynes et al., 2006; Shamay-Tsoory et al., 2009; Larquet et al., 2010).

In brief, the relationship between FI, PCA and dysfunction of executive functions, has been extensively studied (Bechara et al., 2000; Duncan, 2010; Dumontheil et al., 2011). In addition, the link between FI and abstract reasoning (Bunting, 2006; Perfetti et al., 2009) has also been established. However, research that associates this set of variables with social behavior in contexts of interaction with non-pathological samples, are very scarce, even more when it comes to PSA (Roca et al., 2010; Huepe et al., 2011).

The BA10 area could have a role in the ability of self-projection and in PCA, which are needed for other social skills (Buckner and Carroll, 2007). This area plays a central role in the so-called “default network” (Default Mode Network-DMN-) (Buckner and Vincent, 2007; Chen et al., 2008; Ko et al., 2011) and there is now abundant evidence that the DMN has an atypical configuration in subjects exhibiting some form of mental or psychiatric disorder such as attention deficit-hyperactivity disorder-ADHD, depression, Alzheimer's disease, schizophrenia, bipolar disorder, autism spectrum disorders (ASD), among others (Broyd et al., 2009; Minshew and Keller, 2010; Ongür et al., 2010; Pomarol-Clotet et al., 2011; Meda et al., 2012). Several of these disorders represent extreme points within an adaptation continuum. It is therefore possible to hypothesize that individuals belonging to vulnerable contexts, and exhibiting maladaptive behaviors, could present affectation of prefrontal cortex's (FI, SC, and PCA) main functions. In effect, the literature shows that people with problems associated to impulse control, violent behavior, decision taking, morality, empathy, FI, among others, are related with malfunction of prefrontal areas (Raine, 2002; Bechara and Van Der Linden, 2005; Seitz et al., 2006). Similarly, but more radically, we can see similar behaviors in patients with brain injuries in prefrontal cortex. In short, PCA and FI could be linked to aspects of SC such as theory of mind (ToM), handling multiple tasks (multitasking) and frontal functions (Torralva et al., 2007, 2009). Yet, the question still remains whether individuals belonging to vulnerable contexts—with maladaptive behaviors—would show decline of main prefrontal cortex functions and whether the three cognitive components discussed here can predict PSA in this population.

## A preliminary model of FI, SC, and PCA as predictors of PSA

PSA includes multiple dimensions such as social behavior, emotional regulation, and the development of social habits (Bishop et al., 2005; Cox et al., 2010). Psychosocial functioning represents an ecological approach to everyday adaptation as well as a theoretical approach which integrates cognition and emotion (Wilson, 2008). We propose that variables such as FI, SC, and PCA would influence PSA processes, based on the evidence from injured patients, briefly summarized here. We would also expect that PCA will be altered given their link to complex cognitive functions and high-level cognitive skills such as mental state attribution, empathy, and understanding of social contextual cues (Buckner and Carroll, 2007; Ibañez and Manes, 2012). This last issue could be expected from evidence of researches about the role BA10 plays in the understanding of contextual cues. Patients with damages in this area have exhibited significant difficulties to read correctly certain social meanings such as the ability to infer feelings, thoughts and other complex set of functions associated to ToM (Gilbert et al., 2006, 2007).

**Figure 1:**
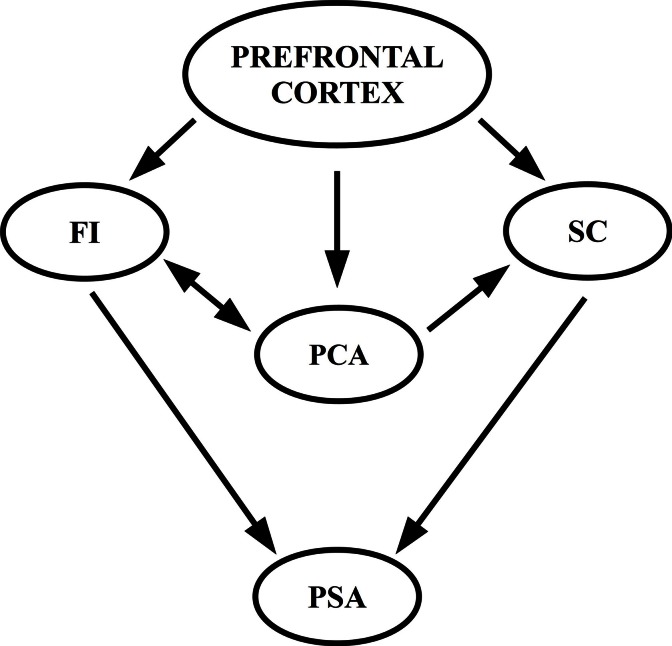
**Tentative model of a framework for the relationship between FI, PCA, SC, and PSA**. Prefrontal cortex, FI, and SC (which includes PCA) predict PSA.

Figure 1 illustrates the relationships among FI, SC, and PCA as a framework to assess PSA individual differences. Arrows indicate the direction of the relationship: FI, SC, and PCA are explained by the prefrontal activation and maturation. In turn, FI and SC predict PSA. Finally, PCA shows an indirect effect mediated by the effect of SC on PSA.

We hypothesize that the degree of PSA that people show in vulnerable social contexts would be partly explained by the level of performance exhibited on FI and SC tasks. SC, in turn, would depend on PCA levels. Previous research supports each of these relationships separately (Gilbert et al., 2006, 2007; Torralva et al., 2007, 2009; Roca et al., 2010, 2011; Huepe et al., 2011). However, how these relationships could predict PSA is a matter of further research.

This framework would help to design empirical models of individual differences of these variables in vulnerable social contexts. Preliminary evidence supports this assertion. For instance, children under vulnerability with better social adaptation have high levels of FI (Huepe et al., 2011). It is also known that people under unfavorable living conditions, have a better PSA when they have high level cognitive skills, facilitating a better social adaptation (Flores et al., 2005; Cicchetti and Blender, 2006).

According to this tentative model, we specifically expect to find that higher levels of FI are associated with PSA. Moreover, a good performance on PCA and SC, positively linked to PSA, is also expected. Indirect evidence from Roca et al. (2011) has suggested that FI predicts the performance on different tests (executive functions and SC) in a group of frontal patients with lesions in BA10 (associated with PCA) when compared to a group of frontal patients (non-BA10), and a group of healthy controls. This evidence shows the importance of BA10 in SC. Patients with lesions in BA10 exhibited lower performance in SC tests and not in ecologically executive functions. Hence, it can be established that these components would be networks that, anatomically and neurally, to some extent are independent, although data is not conclusive on the extent of the kind of association among these components. Thus, we suggest a particular pattern of effects of these variables in our tentative model.

Complex modern societies demand a strong capacity for social adaptation. Bullying and violence, addictive behavior, mental health impairments, and other social behaviors are strongly linked to quality of life. Current agenda includes discovering the processes whereby individuals at high risk do not develop maladaptive or pathological behaviors. Resilience, defined as the accomplishment of competences regardless of significant adversity (Cicchetti and Blender, 2006), could be an example of the implications of social adaptation. Therefore, the framework presented would help to better clarify the psychosocial factors related to how resilience works, by evaluating cognitive protective factors that could be contributing to social adaptation in vulnerable populations.

Our model proposes a straightforward association between levels of FI, PCA, and SC regarding PSA. To prove that certain cognitive abilities have a major impact on PSA is crucial both from a scientific and a political point of view, especially in countries where inequality limits the opportunities of development and academic performance (Lutha and Cicchetti, 2000; Salas et al., 2010). The influence of cognitive functions related to social adaptation may represent a contribution by orienting changes in policy regarding the possibilities of development and intervention. This would be especially relevant in populations suffering from poverty, drug abuse, violence, among others, offering a background to design cognitive interventions that are socially and contextually focused.

In brief, assessing the effects of FI, PCA, and SC would be crucial for understanding the different levels of PSA in vulnerable contexts. This kind of studies would favor a multiple-level-analysis viewpoint in order to design and evaluate interventions that aim at recognizing outcomes related to resilience, cognitive changes, and social adaptation, in persons facing significant adversity (Lutha and Cicchetti, 2000). Neurocognitive markers would help to enlighten the impact of cognitive functions on SC (Ibáñez et al., 2009; Ibañez et al., 2012). Further empirical development of this framework would promote possible future forms of social intervention based on the theoretical and empirical co-construction of tools provided by social neuroscience, neuropsychology, and social psychology.
